# Post traumatic stress disorder and co-morbid psychological disorders after Palestinians’ home demolition: a comparative study

**DOI:** 10.3389/fpsyt.2024.1443374

**Published:** 2024-09-27

**Authors:** Adnan Lutfi Sarhan, Kamal Jarrar, Sameeha Atout, Walaa Masri

**Affiliations:** ^1^ Faculty of Medicine and Health Sciences, An-Najah National University, Nablus, Palestine; ^2^ Department of Data Science and Applied Statistics, Birzeit University, Birzeit, West Bank, Palestine; ^3^ Biotechnology Institute, Ankara University, Ankara, Ankara, Türkiye

**Keywords:** PTSD, home demolition, Palestine, anxiety, depression, stress

## Abstract

**Background:**

Home demolition is one of the issues that the Palestinian community faces as a result of Israeli procedures that can impact Palestinian mental health. This study aimed to measure the prevalence of Post-Traumatic Stress Disorder (PTSD), depression, anxiety, and stress among Palestinian citizens whose homes were demolished.

**Materials and methods:**

A comparative cross-sectional design was adopted using a purposive sample of home demolished versus not home demolished Palestinian people. The Impact of Event Scale-Revised (IES-R) and the Depression and Anxiety Stress Scales (DASS) were used to assess the participants' levels of PTSD, depression, anxiety, and stress. The Statistical Package for the Social Sciences, Version 25, was applied to the statistical analysis.

**Results:**

This study showed that PTSD levels among people whose homes were demolished are significantly high, with a mean of 3.2, which could be indicative of a clinical concern. However, the PTSD level in the comparison group had no significance, with a mean of 1.48. Stress, depression, and anxiety levels were represented as 32.71, in their means, 32.61, and 32.08, respectively, among home-demolished people, compared to stress 18.46, depression 15.87, and anxiety 13.06 among the non home demolished group.

**Conclusions:**

This study is one of the few that sheds light on one of the disadvantaged groups who suffer from home demolition and the severe mental problems that affect them, including PTSD, stress, depression, and anxiety). Furthermore, many related risk factors were studied in this research. As a future recommendation, further research is needed in this field, especially among disadvantaged groups. Stakeholders need to take action to improve the health system in Palestine.

## 1 Introduction

Demolition of homes is a hard and powerful experience that affects all family members and puts them under psychological, social, and financial pressure (1). People are mostly affected by traumatic and threatening events on a psychological level. According to the World Health Organization (WHO), the number of people with mental and psychological disorders is likely to rise in a population that has been occupied for a long time, does not feel safe, has trouble moving around, and has had their human rights violated, including being forced to move (2). Similar to the experiences of university students in Mosul, Iraq, who faced severe mental health challenges due to prolonged conflict and violence (3), Palestinian populations have been subjected to sustained trauma from military actions, including home demolitions. Both contexts highlight the severe impact of war atrocities on mental health, particularly the prevalence of Post Traumatic Stress Disorder (PTSD). These demolition procedures might lead to various negative consequences, including disruptions among family members, culture and identity loss, also problems in mental health outcomes such as stress, anxiety, depression, and PTSD (4). The ongoing threat of home demolitions creates an environment of chronic stress and uncertainty. Living with the constant fear that one’s home could be destroyed at any moment prevents individuals from achieving psychological stability and security, leading to heightened and persistent mental distress. This sudden and violent disruption to life can lead to severe psychological trauma, including PTSD, depression, anxiety, and chronic stress. The personal nature of losing one’s home, often under hostile and traumatic circumstances, exacerbates the mental health impact compared to other stressors associated with prolonged conflict. In comparison to the broader effects of nearly 70 years of occupation, the specific and personal impact of housing-related threats like home demolitions presents a unique and acute challenge to mental health (5).

PTSD can be defined as a mental health disorder caused by witnessing or experiencing terrifying events. It may occur instantly or weeks, months, or even years later (6). The most frequent symptom of PTSD is re-experiencing the traumatic event. It occurs when an individual vividly and uncontrollably relives the traumatic incident through flashbacks, nightmares, recurrent and upsetting pictures or sensations (7). One study conducted in the Gaza strip on children whose homes were demolished versus children whose homes were not demolished or blown up. The outcome was that children exposed to bombardment and home demolition had higher rates of mental disorders such as fear and PTSD than controls (4, 8, 9). Even though there are very few studies about home demolitions among Palestinians, Palestinian society still lacks research on the prevalence of PTSD among those whose homes were destroyed and demolished (10).

Another mental health outcome is depression, which is a mood or emotional state that, according to psychology, is characterized by feelings of guilt or poor self-worth, as well as a diminished capacity to enjoy life (11). Depressed people suffer from sadness, hopelessness, or pessimism; diminished self-worth and increased self-depreciation; a decrease or loss of enjoyment in routine activities; diminished energy and vitality; slowness of thought or action; loss of appetite; and disturbed or unrestful sleep (12). A study done in 2021 showed that both Palestinians whose homes were demolished and those whose homes were constantly threatened with being destroyed shared a common experience of depression (4). Both adults and children suffered psychologically from home demolitions, including depression. Additionally, one study found that adults who had personally experienced home demolitions scored higher on a depression scale than those who had only witnessed (13). It was noticed that Palestinian children, whose homes had been demolished, as well as their loss of sense of safety and confidence, worked on changes in feelings and feelings that cause frustration, despair, and depression, which are difficult to treat quickly (14).

Also, anxiety is a suspected mental health outcome characterized by an uncontrollable, continuous, unpleasant, and protracted state of negative effects that are characterized by the anxious expectation of an unanticipated and unavoidable future danger and is accompanied by bodily tension and a continuing state of enhanced vigilance (15). Moreover, anxiety can manifest as a normal reaction to stress, but when it becomes excessive, persistent, or out of proportion to the situation, it may develop into an anxiety disorder, impacting daily functioning and quality of life (16). Anxiety accompanies physical symptoms like sweating, trembling, dizziness, or an increased heartbeat (17). Although limited research has been conducted to estimate anxiety levels in Palestine as a traumatized society or to correlate anxiety with home bombardments, all of the few studies conducted agree on the high anxiety levels among various groups, including children, adolescents, parents, etc.). Interestingly, two comparative studies have reported higher anxiety levels in people who have not experienced home demolition directly than the ones who were exposed to home demolition (8, 18). Also, Palestine has recorded the highest anxiety levels among Middle Eastern countries (19). Moreover, two studies were conducted in the Gaza Strip after the second uprising, which showed severe anxiety levels in the selected samples (20, 21). It is important to check carefully for anxiety because between 2% and 4% of the general population shows enough symptoms to be labeled as having an anxiety disorder (22, 23).

The last mental health outcome is stress, which is defined as a state in which a person is annoyed and worried by an unpleasant challenge they cannot avoid (24). Previous studies have demonstrated that children who are exposed to serious and continuing dangers to their safety, such as poverty or violence, frequently without the protective qualities of their surroundings, experience toxic stress (25, 26). The effects of home demolition have been experienced by children, women, and adolescents in various psychological aspects (27, 28). Furthermore, sleeping problems and a decline in mental health have been shown by children of families after home demolition (4, 29).

The present research aims to assess the prevalence of PTSD, depression, anxiety, and stress among Palestinians who have experienced home demolitions compared to those who have not. By using structured instruments and a comparative cross-sectional design, this research seeks to provide a clearer understanding of the mental health impacts of home demolitions in the context of the ongoing conflict.

## 2 Methods

### 2.1 Participants

A comparative cross-sectional design was adopted to investigate the prevalence of PTSD, depression, anxiety, and stress in people with demolished homes versus those who did not in the West Bank and Jerusalem-Palestine, between October and December 2022.

The home demolished sample consisted of 51 families, among which 93 participants were interviewed, including more than one person from the same family, compared to the 346 participants in the comparison group. The judgment sampling technique, also known as purposive sampling, was used, which involves selecting participants consciously based on a certain trait, which is that the home was demolished by the Israeli occupation and not by any other cause.

### 2.2 Procedure

The data were collected through structured interviews with the participants. The researchers visited the demolished homes of participants in their homes after contacting them by telephone to fix an appointment. The face-to-face interview with family members took 20-30 minutes at a convenient place.

Due to the lack of data on families whose homes have been demolished at the Palestinian statistical center, the study adopted members of families whose homes have been demolished within the last two years. Members who did not agree to participate were excluded. The comparison group included members of families who did not have home demolition during the same period (2020 – 2022). Anyone from the selected groups who agreed to and signed the consent form were included in the current study. Also, anyone who disagreed was excluded.

### 2.3 Measures

The first part of the questionnaire collected demographic information through individual interviews, including gender, age, and monthly income. During the interviews, the researches introduced their self and started the interview by engaging the participants into a rapport trustful relationship. The trust relationship was enhanced due to that the participants considered the researchers as supporter persons after the time of home demolition. Regarding the non-demolished homes group, also, they selected according to their agreement to participate. The introductory qualitative interview intended to gather the demographic data and enabling the participants to use the debriefing process to drain their psychological concerns toward the traumatic event of home demolition.

Two scales were used based on previous reviews and literature: the PTSD scale, Impact of Event Scale-Revised (IES-R) and the Depression Anxiety Stress Scale (DASS-42), which were used to assess the participants’ levels of PTSD, depression, anxiety, and stress.

Impact of Event Scale-Revised (IES-R) is a 22-item self-report measure that assesses subjective distress caused by traumatic events, including hyperarousal symptoms. The tool, not diagnostic for PTSD, is an appropriate instrument to measure the subjective response to a specific traumatic event (30). After identifying a particular stressful life event, respondents were asked to rate how concerned or troubled they were by each “difficulty” described within the previous seven days.

Assessing measurement invariance using IES-R rated for each item from 0 (“not at all”) to 4 (“Extremely”) on a 5-point scale. The intrusion, avoidance, and hyperarousal subscales of the IES-R can also be used to construct subscale scores. We used the scale with its total score including the IES-R subscores, which range from 0 to 88. 24-32: Clinically, people with scores this high who do not have complete PTSD will at least have some of the symptoms of partial PTSD (31). The optimal cut-off for a likely diagnosis of PTSD is between 33 and 38, 39 or higher, even ten years after an impact event, this level will suppress the function of your immune system (32).

The Arabic version of the scale was translated from English, revealing a high acceptable value of coefficient alpha, which was found to be 0.90 and 0.93 (33).

Depression, Anxiety, and Stress Scales (DASS-42) is the third part of the interview consisted of 42 self-reported items that assess three major psychological dimensions, namely depression, anxiety, and stress, with just one administration of the DASS (34). The essential function of the DASS is to assess the severity of the core symptoms of depression, anxiety and stress. In DASS, each item indicates a distressing emotional symptom. To emphasize states over traits, each of these is assessed on a four-point Likert scale based on the frequency or severity of the participants’ experiences throughout the previous week. These scores varied from 0 (“did not apply to them at all”) to 3 (“applied to them a significant lot or most of the time”) (35). Confirmatory factor analysis indicates that the Arabic DASS discriminates between depression, anxiety, and stress to a lesser amount than the English DASS, besides all 42 items’ factor loadings matched those of the English DASS, proving that the items’ translation and adaptation were accurate (36). The internal reliability of the DASS-42 subscales for anxiety, depression, and stress was assessed using Cronbach’s alpha. Alpha values were 0.888 for the depression scale, 0.866 for the stress scale, and 0.833 for the anxiety subscale (36). Subscales’ item-internal consistency is good (Cronbach’s alpha values above 0.70) (37).

### 2.4 Data analysis

The statistical analysis was conducted using IBM SPSS version 25 (SPSS Inc., Chicago, IL, USA) (38). All required analyses were carried out, and the tests’ significance threshold was set at 5%. Initially, parametric tests were conducted since the results of the normality tests indicated that the data were roughly normally distributed (Kolmogorov-Smirnov p < 0.005). The study variables underwent descriptive analyses, which involved the computation of means, standard deviations, and frequencies. Bivariate correlation analysis (Pearson’s r) was performed for all variables to observe their association.

Three blocks of variables were used in a hierarchical multiple regression to test the predictions. In the first step, the dependent variables were stress, anxiety, and depression, while the predictors were gender, age, and monthly income. In step 2, stress, depression, and anxiety were predicted using the following factors: gender, age, monthly income, and PTSD. The results of the study showed that gender (β = 0.404; ** p <.01) and PTSD (β = 0.779; ** p <.01) positively predicted stress. Conversely, stress was not predicted among those whose homes were not demolished, but it was adversely predicted among those whose homes were destroyed (β = -0.392; ** p <.01).

The findings showed that PTSD (β = 0.768; ** p <.01) and gender (β = 0.382; ** p <.01) positively predicted anxiety, respectively. Furthermore, age was found to be a negative predictor of anxiety (β = -0.352; ** p <.01). Ultimately, the study’s findings revealed that gender and PTSD both positively predicted depression (β = 0.396; ** p <.01) and (β = 0.816; ** p <.01), respectively. Age was found to have a negative correlation (β = -0.305; ** p <.01).

### 2.5 Ethical considerations

This study was carried out in accordance with the guidelines of the Declaration of Helsinki. The study received ethical approval (reference number Med. Oct. 2022/32) from the Institutional Review Board (IRB) of An-Najah National University. Also, every individual participant in the study gave informed consent.

## 3 Results

**Table 1**. illustrates the demographic characteristics of the study sample including home demolished (94) and non-home demolished citizens (345). The majority of home demolished are males. Also, the highest percentage was of a group of 46-65 years old. Regarding the non-demolished homes group, the majority of the participants were females from the age of 26-45 years old.

**Table 1:** Demographic characteristics of the study sample.

Variable		N (%)	Mean	S.D	Total
Home demolished (N=94)					
Sex	Male	54 (57.4)	1.426	0.497	94
	Female	40 (42.6)			
Age	< 15Y	24 (25.5)	2.798	1.292	
	15-25Y	13 (13.8)			
	26-45Y	19 (20.2)			
	46-65Y	34 (36.2)			
	>65 Y	4 (4.3)			
Non-home demolished (N=345)					
Sex	Male	143 (41.4)	1.586	0.493	345
	Female	202 (58.6)			
Age	< 15Y	0	3.003	0.929	345
	15-25Y	113 (32,8)			
	26-45Y	153 (44.3)			
	46-65Y	44 (12.8)			
	>65 Y	35 (10.1)			

**Table 2**. illustrates the results of descriptive statistics, showing high mean scores for PTSD (3.20), stress (32.71), depression (32.61), and anxiety (32.08) among home demolished people.

**Table 2:** Descriptive statistics of dependent variables regarding home and non-home demolished people.

Variable		Mean	S.D	Min	Max	Range	Skewness	Kurtosis
PTSD	**Home demolished** **(N=94)**	3.2013	0.67796	0.27	4.00	3.73	-1.464	2.923
Stress		32.7128	7.11295	10.00	42.00	32.00	-1.080	0.712
Depression		32.6170	7.54500	9.00	42.00	33.00	-1.116	0.581
Anxiety		32.0851	7.40773	10.00	42.00	32.00	-0.908	0.240
PTSD	**Non Home demolished** **(N=345)**	1.4807	1.07369	.00	3.56	3.56	-.120	-1.237
Stress		18.4638	9.96065	.00	41.00	41.00	.107	-.752
Depression		15.8725	9.66900	.00	42.00	42.00	.434	-.436
Anxiety		13.0696	8.43655	.00	41.00	41.00	.670	.054

SD, Standard Deviation.

When comparing these results with those of non-demolished homes, the findings indicate that the residents of non-demolished homes experienced lower levels of PTSD (1.48), stress (18.46), depression (15.87), and anxiety (13.06).

The findings of correlational analyses are present in **Table 3**. Among home demolitions, the results show that there is a positive correlation between PTSD and stress (r = 0.87, p <.01), depression (r = 0.85, p <.01), and anxiety (r = 0.83, p <.01). Moreover, stress is positively correlated with depression (r = 0.87, p <.01) and anxiety (r = 0.89, p <.01). Also, the correlational results of non-home demolished people were positively correlated, PTSD and stress, (r = 0.377, p <.01), depression (r = 0.378, p <.01), and anxiety (r = 0.494, p <.01). In addition, stress is positively correlated with depression (r = 0.811, p <.01) and anxiety (r = 0.691, p <.01) among non-home demolished people. And depression is positively correlated with anxiety.

**Table 3:** Correlations among study variables for home demolished and non-demolished people.

Measures		*1*	*2*	*3*	*4*
1. PTSD	**Home demolished**		0.87**	0.85**	0.83**
2. Stress				0.87**	0.89**
3. Depression					0.88**
4. Anxiety					
1- PTSD	**Non Home demolished**		0.377**	0.378**	0.494**
2- Stress				0.811**	0.691**
3- Depression					0.702**
4- Anxiety					

Significant correlation, **.

The findings in **Table 4** present hierarchical regression analyses predicting stress, anxiety, depression, and PTSD based on sociodemographic variables (gender, age, monthly income) in step 1.

**Table 4:** Hierarchical regression analysis for variables predicting depression, anxiety, and stress.

Demolished homes				Non demolished homes				
Variable	B	SEB	β	R2	B	SEB	β	R2
*Stress*								
*Step1*								
Gender	5.783	1.221	0.404**	0.357	0.488	1.126	0.024	0.005
Age	-3.224	0.702	-0.392**		0.326	1.120	0.015	
Monthly income	1.591	1.684	0.080		-4.488	3.629	-0.068	
*Step2*								
Gender	1.370	0.811	0.096	0.771	0.469	1.042	0.023	0.150
Age	-0.946	0.458	-0.115**		0.413	1.111	0.019	
Monthly income	0.625	1.013	0.031		-5.556	3.363	-0.084	
PTSD	8.174	0.644	0.779**		3.531	0.464	0.381**	
*Anxiety*								
*Step1*								
Gender	5.689	1.325	0.382**	0.303	0.271	0.953	0.016	0.007
Age	-3.017	0.761	-0.352**		0.236	1.016	0.013	
Monthly income	0.326	1.826	0.016		-4.494	3.071	-0.080	
*Step2*								
Gender	1.157	0.959	0.078	0.705	0.250	0.827	0.015	0.254
Age	-0.677	0.541	-0.079		0.332	0.881	0.019	
Monthly income	-0.666	1.197	-0.032		-5.677	2.668	-1.101**	
PTSD	8.394	0.761	0.768**					
**Variable**	**B**	**SEB**	**β**	**R2**	**B**	**SEB**	**β**	**R2**
*Depression*								
*Step1*								
Gender	6.007	1.371	0.396**	0.280	0.444	1.092	0.023	0.008
Age	-2.666	0.788	-0.305**		0.335	1.163	0.016	
Monthly income	1.120	1.890	0.053		-5.490	3.518	-0.086	
*Step2*								
Gender	1.103	0.927	0.073	0.734	0.426	1.010	0.020	0.153
Age	-0.135	0.523	-0.015		0.420	1.076	0.020	
Monthly income	0.046	1.158	0.002		-6.531	3.257	-0.102**	
PTSD	9.084	0.736	0.816**		3.441	0.450	0.382**	

Significant correlation, **.

In step 2; the gender, age, monthly income, and PTSD were used to predict stress, depression, and anxiety. The study findings revealed that stress was positively predicted by gender (β = 0.404; ** p <.01) and PTSD (β = 0.779; ** p <.01). On the other hand, negatively predicted by age (β = -0.392; ** p <.01) among demolished homes people, while stress was not predicted among non-home demolished.

Regarding anxiety, the results found that it was positively predicted by gender (β = 0.382; ** p <.01) and PTSD (β = 0.768; ** p <.01), respectively. Also, the results revealed that anxiety was negatively predicted by age (β = -0.352; ** p <.01). Finally, the study results found that depression was positively predicted by gender and PTSD (β = 0.396; ** p <.01) (β = 0.816; ** p <.01), respectively. While negatively predicted by age (β = -0.305; ** p <.01).

The multicollinearity statistics of regression and Variance Inflation Factors (VIF) were tested to reveal that when two or more predictor variables have a high degree of correlation with one another and do not contribute distinct or independent information to the regression model. The fitting and interpretation of the regression model may be complicated if there is a significant degree of correlation between the variables.

The VIF values for the predictor variables in this study (1-2) are not greater than 5, which indicates that multicollinearity will not be a problem in the regression model. This indicated that p-values in the regression output are likely reliable.

## 4 Discussion

The primary objective of this study is to assess the prevalence and severity of mental health disorders, particularly PTSD, depression, anxiety, and stress, among Palestinians who have experienced home demolitions compared to those who have not. This study is guided by several key hypotheses. First, it is hypothesized that individuals who have experienced home demolitions will exhibit significantly higher levels of these mental health disorders than those who have not. Additionally, the study posits that the psychological impact of home demolitions is more severe and immediate compared to the broader, ongoing stressors of occupation and conflict. Furthermore, it is hypothesized that sociodemographic factors, such as age, gender, and location, may moderate the relationship between home demolitions and mental health outcomes, with certain groups being more vulnerable to severe psychological distress. Lastly, the study hypothesizes that chronic stressors, like the continuous threat of home demolitions, exacerbate the impact of previous traumatic experiences, leading to a cumulative effect on psychological well-being.

In our research sample, participants who had their homes demolished had an overall PTSD mean score of 3.2, indicating PTSD as a clinical concern. One study showed similar high results of PTSD among Lebanese and Gazan participants who had their homes demolished (39). Moreover, the results were consistent with other studies in the area where the participants had suffered from military violence (40, 41). The prevalence of PTSD and other psychological disorders among Palestinians whose homes were demolished was consistent with findings from other conflict zones such as Iraq. For instance, Arafat et al. reported a 12% prevalence of PTSD symptoms, with higher rates of distress associated with direct exposure to traumatic events such as house raids and property damage (3). This comparison underscores the pervasive impact of conflict-related trauma across different populations. Almost a quarter of the current study sample showed symptoms of PTSD. These results agree with another study where 25.7% of the study sample demonstrated PTSD symptoms (41).

Among the home demolition group, PTSD levels in females were higher than that in males. These results were consistent with other studies (41–43), however, one study showed that gender is not considered a risk factor of PTSD (44). In our study, the overall PTSD symptoms were higher in females, while another study found that only the intrusion results were higher in females compared to the other scales, which were almost the same (43). The difference between these results might be due to the type of trauma that led to PTSD development. Going through direct military violence and staying under the threat of missiles, random shootings, and shelling might be harder than witnessing home demolitions. Thus, survivors of military violence keep reliving such events over and over again in their minds. Moreover, in Palestinian society, one of the reasons for this might be masculinity issues, where males always try to hide their feelings to appear strong and able to protect themselves and their families (44, 45). This was very obvious during the data collection process, where males were trying to show that they are not affected by the situation as a kind of resistant procedure.

In addition, a study among Palestinian Bedouin women who are at risk of losing their homes found a higher depression than those who are not at risk (46). Another study done on the Bedouin community showed that the demolition of a home causes double stress for adolescents compared to those whose homes are under threat of demolition (29). These results indicate the importance of homes, which represent the protective shield against the world, and how destroying them participates in developing PTSD and other mental disorders (43).

Like PTSD, a variation in the levels of anxiety, depression, and stress was recorded between genders. Females have always recorded higher levels of mental disorders in both demolished and non-demolished groups. These results are consistent with other studies (41, 44), which can be related to the emotional state of women compared to men (47). However, in a study that was done on Palestinian children, no differences in anxiety levels were found between genders (41). This can be explained by the children’s physiological norms that have no difference between genders, and emotional states that begin to develop after puberty (41).

Age plays an important role in the development of mental disorders (stress, anxiety, depression, and PTSD). In the demolished group, children younger than 15 years old had the highest scores of PTSD where the symptoms were high enough to suppress the immune system’s functioning. These results were consistent with studies in which the prevalence of PTSD and other mental disorders is high among children (44, 45, 48, 49). This could be due to the vulnerability, fragility, and sensitivity of children to external stressors (50). On the contrary (44), indicated that older age is a risk factor for the development of mental disorders.

Interestingly, among the group of home demolitions, family income did not have any effect on the levels of PTSD, stress, anxiety, or depression. However, one study showed that low income is considered a risk factor for developing PTSD and mental disorders (44). In the case of Palestinian society, this difference can be attributed to the huge effect of this type of trauma (home demolition) that can barely be recovered by either high or low levels of family income.

Compared to the comparison group, stress, anxiety, and depression scores were significantly higher among the demolition group. Even though Palestinians live under special circumstances of continuous military violence, the levels of stress, anxiety, and depression have not exceeded the moderate level in the control group. However, Palestinians who experienced home demolition have recorded severe levels of stress and extremely severe levels of both depression and anxiety (41).

Two major hypotheses were concluded from our results. First, there is a positive relationship between PTSD and other mental health outcomes (stress, depression, and anxiety). Having PTSD increases the potential of having one or more of the other mental health disorders. These results are consistent with the results of Astitene and Barakat (51). Second, among the home demolished, PTSD and certain sociodemographic factors (gender and age) can serve as predictors of stress, anxiety, and depression levels. Stress, anxiety, and depression were shown to be positively predicted by both PTSD and gender factors, i.e., having PTSD and being female increase the chances of having one of the mental health disorders. On the other hand, age was indirectly related to stress, anxiety, and depression, i.e. The younger the age, the higher the possibility of developing stress, anxiety, or depression. These results are similar to the findings of Abu-El-Noor (52). However, Khamis et al. showed that age has no association with depression or anxiety. Furthermore, monthly income, which in our results showed no association with any of the mental health disorders, was a negatively predictive variable for depression (53).

The process of conducting this research has faced some obstacles and limitations. First, although the sample size was relatively small, reaching out to the participants was challenging due to the political situation in Palestine, as there were road restrictions and military barriers along the roads, especially during the period when this research was conducted. Moreover, some families in the sample were residing in areas under total Israeli control, such as East Jerusalem, which made access to the area a complicated process. Second, due to the masculinity culture among male Palestinians, many of them refused to express their true feelings in front of us as a trial to show strength. Many of them pretended to be strong as a means of occupation resistance, they thought of expressing their feelings as if this breaks their pride. Furthermore, a trend was noticed among the participants who had their homes demolished, as many of them were expecting a kind of recovery or compensation for their loss, or they thought that participating in the research might allow them to be recovered in the future (thinking that this research is something related to the government). Some of the potential participants, especially those who were from East Jerusalem, were afraid of participating, thinking that this would expose them to further Israeli harassment.

Having a purposive sample means that our results are more prone to bias and restricting its generalization (54). Furthermore, the control group did not include any children, while around a quarter of the participants in the home-demolished group were children. This makes it difficult to make comparisons and conclusions about this vulnerable group.

This study is one of the few that sheds light on one of the disadvantaged groups that suffer from home demolition. This research highlights the severe mental problems that affect the previous inhabitants of the demolished homes, including stress, anxiety, depression, and PTSD. Our results show many risk factors that might assist in developing mental disorders among the survivors, which in turn might help clinicians make early interventions in these cases. As future recommendations, further research is needed in this field, especially among disadvantaged groups. Stakeholders need to take action in improving the health system in Palestine so that it can deal professionally and carefully with the survivors of home demolition and other military actions. Additionally, clinical centers specialized in providing mental health support for the victims, survivors of military actions, and disadvantaged groups are needed to be established and activated in Palestine.

## Data Availability

The original contributions presented in the study are included in the article/supplementary material. Further inquiries can be directed to the corresponding author.
